# The Ecdysis Triggering Hormone System, via ETH/ETHR-B, Is Essential for Successful Reproduction of a Major Pest Insect, *Bactrocera dorsalis* (Hendel)

**DOI:** 10.3389/fphys.2019.00151

**Published:** 2019-03-18

**Authors:** Yan Shi, Tian-Yuan Liu, Hong-Bo Jiang, Xiao-Qiang Liu, Wei Dou, Yoonseong Park, Guy Smagghe, Jin-Jun Wang

**Affiliations:** ^1^Key Laboratory of Entomology and Pest Control Engineering, College of Plant Protection, Southwest University, Chongqing, China; ^2^Academy of Agricultural Sciences, Southwest University, Chongqing, China; ^3^International Joint Laboratory on China-Belgium Sustainable Crop Pest Control Between Southwest University in China and Ghent University in Belgium, Chongqing, China; ^4^Department of Entomology, Kansas State University, Manhattan, KS, United States; ^5^Department of Plants and Crops, Faculty of Bioscience Engineering, Ghent University, Ghent, Belgium

**Keywords:** *Bactrocera dorsalis*, ecdysis-triggering hormone, juvenile hormone, 20-hydroxyecdysone, vitellogenin, reproduction, RNAi

## Abstract

Ecdysis triggering hormone (ETH), released by the Inka cells, is a master hormone in regulating the ecdysis process in insect. Here we investigated the presence and role of the ETH signaling in the female adult of the oriental fruit fly, *Bactrocera dorsalis* (Hendel) that is one of the most important invasive pest insects in agriculture worldwide. In the female adult, ETH was confirmed in the Inka cells at the tracheae by immunostaining and also *in vitro* exposure to ETH stimulated the isolated corpora allata of adult in activity. Then we prepared cDNA of females at 0, 5, 10, 15, and 20 days after adult eclosion, and RT-qPCR showed that the expression pattern of *ETH* and its receptor *ETHR-B* started from a peak at the day of adult eclosion (day 0), then dropped to basal levels and increased again between day 10 and 15 which is also the period corresponding to ovary growth. In contrast, *ETHR-A* was absent with Ct values of >33. The expression patterns of the ecdysteroid-producing Halloween genes *Spook* and *Shade*, and the vitellogenin genes *Vg1*, *Vg2*, and *Vg3* co-occurred with peak levels at days 10–15, and also juvenile hormone acid methyltransferase (*JHAMT*) showed increased levels on day 15. Further in RNAi assays to better understand the role of ETH and ETHR, dsRNA was injected to adult and this led to a respective decrease in expression of 62 and 56% for *ETH* and *ETHR-B*, while *ETHR-A* stayed absent with Ct values of 33. In these RNAi-females, there was an apparently decreased expression for *JHAMT* and *Vg2*, together with a significant decrease of the JH titer and egg production. Injection of the JH mimetic methoprene could rescue *Vg2* expression and egg production. Upstream, in ds*ETH*/ds*ETHR*-injected females, 20-hydroxyecdysone (20E) injection rescued the transcriptions of *ETH* and *ETHR* and also egg production. In summary, our results shed more light on the pivotal role that the ETH peptide hormone and its receptor ETHR-B play an essential role in the reproduction of the female adult of *B. dorsalis*, via the regulation of JH and vitellogenin, which are controlled by a pulse of 20E.

## Introduction

The oriental fruit fly, *Bactrocera dorsalis* (Hendel) (Diptera: Tephritidae) is a quarantine polyphagous pest and one of the most destructive pest of fruit and vegetable industries, wide distribution and rapid invasiveness because the females show a very highly reproductive capacity (Clarke et al., 2005; Stephens et al., 2007).

The insect reproductive system is regulated by hormones produced in the neuroendocrine system. Ecdysis triggering hormone (ETH) is a small C-terminally amidated peptide hormone, and when released from the Inka cells into the hemolymph, it acts as a command chemical to trigger a behavior sequence critical for shedding of the exocuticle in insects at the larval stages (Park et al., 1999; Zitnan et al., 2003; Li and Adams, 2009; Roller et al., 2010). Typically, deletion or knocking down expression of the ETH gene results in ecdysis-related deformities and lethal phenotypes (Park et al., 2002; Lenaerts et al., 2017; Shi et al., 2017). Previous observations found that Inka cells, the sole source of ETH, persist into the adult stage implied the possible reproduction and courtship memory functions for this peptide system (Park et al., 2002; Lee et al., 2017; Meiselman et al., 2017).

On the regulation upstream of ETH, previous studies demonstrated that 20-hydroxyecdysone (20E) regulates synthesis and release of ETH and expression of its receptor ETHR in the larval stages of moths, mosquitoes, desert locust and the adult of *Drosophila* (Zitnan et al., 1999; Zitnan et al., 2007; Li and Adams, 2009; Lenaerts et al., 2017; Meiselman et al., 2017). Because 20E is of major physiological and reproductive relevance (Parthasarathy et al., 2010a; Schwedes and Carney, 2012; Meiselman et al., 2018), we measured whether 20E influences the gene expressions of *ETH/ETHR* during the female adult stage. We hypothesize here that 20E regulates ETH signaling and in turn the high reproduction capacity of *B. dorsalis*.

On the function of ETH itself, in the yellow fever mosquito *Aedes aegypti*, the peptide was found to work as an allatotropin with an activation of juvenile hormone (JH) acid methyltransferase (JHAMT) (Areiza et al., 2014). Previous studies also detected *ETHR* transcripts in the corpora allata (CA) of *Bombyx mori*, *Manduca sexta*, and *Drosophila melanogaster* (Yamanaka et al., 2008; Areiza et al., 2014; Meiselman et al., 2017). CA is responsible for release of JH which has a prominent role in the regulation of development, reproduction, courtship, and diapause in insects (Jindra et al., 2013; Smykal et al., 2014; Wijesekera et al., 2016; Lee et al., 2017). Therefore, we hypothesize here that ETH regulates JHAMT expression, which encodes JH acid o-methyltransferase and catalyzes a final step in JH synthesis (Niwa et al., 2008), and which in turn controls the JH titer, ovary growth and reproduction in the female adult.

This study is a continuation of our previous study on ETH signaling where we confirmed the role of the ETH/ETHR-A in the ecdysis process in the larval stages of *B. dorsalis* (Shi et al., 2017). Now, we aim to unravel on the presence and function of ETH and its receptors ETHR-A and -B in the female adult stage and the interaction with 20E and JH. This new information will help to understand ovary growth and the high reproductive success in *B. dorsalis*. Hereto, at first, we investigated for the presence of ETH and the adult Inka cells at the tracheal system in the female with an immunochemical assay, and then for its stimulatory role on the CA with isolated CA cultures from females under exposure of ETH peptide. Subsequently, we prepared a cDNA library with female adults at different time points after adult eclosion, which are corresponding to the growth of the ovary in the female adult. To document on the presence of 20E, JH, and vitellogenin, we followed the expression of essential marker genes, such as: two Halloween genes *Spook* and *Shade*, which are involved in 20E production; *JHAMT* related to JH production; and three vitellogenin genes (*Vg1*, *Vg2*, *Vg3*) involved in oocyte growth. Subsequent RNAi assays with ds*ETH* and ds*ETHR* were conducted to confirm the functions in JH titer synthesis, ovary growth and egg production. Finally, the rescue experiments with 20E and JH mimetic (methoprene) were carried out to show their position up or downstream of ETH signaling. We believe these new data will help to further unravel the role of ETH and its receptor in the female adult insect. Specifically, if our hypothesis that receptor isoforms may difference in presence and function during the different developmental stages of the same species, is confirmed, then it is unique that the ETHR-B is playing a role in the female adult via JH in ovary growth regulation. While it is the ETHR-A isoform in the larval immature stages that is regulating the ecdysis progress (Shi et al., 2017). In addition, we expect that the ETH signaling is under control of a pulse of 20E. Finally, because this work was done with a world important agricultural pest insect, the oriental fruit fly *B. dorsalis*, it may provide novel insights to pest control through the manipulation of the ETH pathway and/or interactions with commercial 20E and/or JH agonists or antagonists.

## Materials and Methods

### Insect Rearing

All stages of a continuous colony of *B. dorsalis* were maintained as previously described (Shen et al., 2013; Wang et al., 2013). Briefly, *B. dorsalis* were reared in the Insect Molecular Ecology laboratory of Southwest University at 27°C, 70% relative humidity, and a day/night (14:10 h) photoperiod cycle. Adult females oviposited into pinpricked plastic tubes containing fresh orange juice and then eggs were collected. Newly hatched larvae and newly emerged adults were fed with different artificial diets as previously described (Wang et al., 2013).

### Immunohistochemistry and *in situ* Hybridization for ETH Detection

The tracheae of *B. dorsalis* female adult were dissected in chilled phosphate buffered saline (PBS, 0.01 M, pH = 7.4). Immunohistochemistry was conducted as previously described (Jiang et al., 2013) with the antibody (1:1000 dilution) as prepared for the ETH peptide of *D. melanogaster* (DmETH1, DSSPGFFLKITKNVPRLamide) (Park et al., 2002). For *in situ* hybridization, the ETH probes were prepared by the method of asymmetric PCR using a DIG Probe Synthesis Kit (Roche, Mannheim, Germany) and analyzed as previously described method (Shi et al., 2017).

### *In vitro* Bioassay With Isolated CA

We selected 15-day-old female adults and dissected the CA in PBS 1% under a binocular stereoscope. Upon dissection, the CA were immediately placed in a Petri dish (Diameter 90 mm, Thermo Fisher Scientific) and incubated at room temperature in the dark for 60 min in Hank’s balance salt solution (HBSS, pH = 6.7–7.8) containing non-fluorescent acetoxymethyl ester (Fluo-4 AM, 50 μM) and pluronic F-127 (0.1%) following the protocol of the supplier (Life Technologies, Carlsbad, CA, United States). After incubation, the Fluo-4 AM solution was washed out three times with HBSS. Calcium was visualized with a confocal microscope (Zeiss LSM780, Zeiss, Jena, Germany). The excitation wavelength was 488 nm, and exposure time was 1 s. Images were acquired for 1 h continuous from each tissue preparation and ETH (GenScript, Piscataway, NJ, United States) was applied into a bathing media ∼9 min after imaging onset. The volume of applied ETH was 5 μl (ETH was added in ∼9 min after imaging onset). We used a mixture of BdETH1 and BdETH2 for all experiments; 600 nM ETH (600 nM BdETH1 + 600 nM BdETH2) was added to a stagnant bathing bath with a micropipette. The calcium level was represented as relative fluorescence changes (ΔF/F), where F is the baseline fluorescence and ΔF is the difference between the peak fluorescence caused by stimulation.

### Molecular Cloning and Phylogenetic Analyses of *BdJHAMT*

Based on the genome database and transcriptome of *B. dorsalis*^1^, the *BdJHAMT* gene was identified by performing a TBLASTN search for the *JHAMT* homolog in *D. melanogaster*. RNA was extracted and first-strand cDNA prepared as previously described method (Shi et al., 2017). Primers were designed based on the genome data of *B. dorsalis* (Supplementary Table S1). Using high fidelity DNA polymerase PrimeSTAR (Takara), the full open reading frame (ORF) of *BdJHAMT* was amplified. PCR conditions were as follows: 98°C for 3 min, 95°C for 30 s, 60°C for 30 s, and 72°C for 1.5 min with 35 cycles, and finally 72°C for 10 min. PCR product was purified and cloned into a pGEMT Easy vector (Promega, Beijing, China) and sequenced (BGI, Beijing, China). All JHAMT AA sequences were aligned using ClustalX2 software (Larkin et al., 2007) with default settings (Supplementary Table S2). A neighbor-joining tree, gaps/missing date-pairwise deletion, was produced in MEGA 5.0 (Tamura et al., 2011) with 1,000 bootstrap replicates.

### cDNA Library Preparation and RT-qPCR of Marker Genes

We collected virgin female flies on 0, 5, 10, 15, and 20 days after eclosion. At every time point, four biological replicates were performed and each replicate consisted of four adults. Subsequently, the flies were homogenized in Trizol reagent (Invitrogen, Carlsbad, CA, United States) and then RNA was extracted and first-strand cDNA was prepared with PrimeScript first-strand synthesis system (Takara, Dalian, China).

For the RT-qPCR analysis, we selected the ETH signaling genes (*ETH*, *ETHR-A*, and *ETHR-B*) and pathway marker genes (*Shade*, *Spook*, *JHAMT*, *Vg1*, *Vg2*, and *Vg3*). The reactions were performed in duplicate in 384- or 96-well plates on a StepOne system (ABI, Applied Biosystems, Foster City, CA, United States). The 10 μl of each reaction contained 5 μl of SYBR Green Supermix (Novoprotein, Shanghai, China), 3.5 μl of nuclease free water, 0.5 μl of each primer, and 0.5 μl of cDNA sample (∼200 ng/μL). The PCR conditions were 95°C for 2 min, and 40 cycles of 95°C for 15 s and 60°C for 30 s, 95°C for 1 min. This was followed by melting curve analysis (60–95°C). Based on our previous evaluations (Shen et al., 2010), *α-tubulin* (GU269902) was used as a stable reference gene. All data were analyzed with qBase software (Jan et al., 2007).

### RNA Interference (RNAi) Assays

To explore the functions of ETH signaling in female *B. dorsalis*, RNAi bioassays with dsRNA injections were performed. The most unique nucleotide regions of *BdETH* and *BdETHR* were selected to design the specific dsRNA sequences (Supplementary Table S1), and ds*GFP* was used as a negative control. These dsRNAs were synthesized with the Transcript Aid T7 High Yield Transcription Kit (Thermo Scientific, Vilnius, Lithuania). For injections of dsRNA, 10-day-old virgin female flies were injected with 1.5 μg of dsRNA. After 24 or 48 h, whole bodies were homogenized and mRNA samples were extracted using Trizol (Life Technologies). The RNAi efficiency was calculated by RT-qPCR based on four biological repeats.

### High Performance Liquid Chromatography (HPLC) Analysis for JH III Titer

The samples were extracted and the HPLC analysis executed as previously described method with slight modifications (Zhou et al., 2011). Methanol, hexane, acetonitrile, ether and JH III were all purchased from Sigma-Aldrich. Prothorax and head tissues were collected from 100 females (treatment and control) for each sample with three biological repeats. All the tissues were transferred into four 1.5 ml-centrifuge tubes filled with 1 ml of methanol and fully grounded, respectively. Then, the samples were sonicated for 3–5 min using an ultrasonic cleaning bath (model KQ100E; Kunshan Instruments, China). After the addition of 500 μl of hexane, the samples were sonicated for 10 min. The mixed solution was centrifuged at 10,000 rcf for 10 min, and then the upper hexane layer was transferred into a new 2 ml tube. Ultrasound-assisted extraction was repeated five times with hexane. After the end of each sonication, hexane solution was introduced in the centrifuge tube. The combined extracts were evaporated to dryness under a CoolSafe 55-4 (ScanLaf, Labogene, Lynge, Denmark). The residue was reconstituted in 200 μl of acetonitrile and transferred to injection vials (insert for shell vial), and analyzed by an Agilent 1260 HPLC (Agilent Technologies Inc., Germany) to determine the concentration of JH III. Samples were eluted through a Zorbax 300SB-C18 column (4.6 mm × 150 mm) with a solvent mixture of 75% methanol and 25% water at a flow rate of 0.6 ml/min and injection volume of 10 μl. The column thermostat was maintained at 25°C.

### Effect of 20E on the Expression of *BdETH* and *BdETHR*

A 5 μg/μl-stock solution of 20E (Sigma-Aldrich, St. Louis, MO, United States) was prepared in 95% ethanol. The 5-day-old females were injected with a 20E solution at doses of 0.5 μg/fly. The control was injected with an equivalent volume of ethanol. Four injected females were randomly selected at 8 h after injection, and total RNA was isolated to analyze relative expression levels of *BdETH* and *BdETHR* by RT-qPCR as described above.

### Observation of Egg Numbers

Newly eclosed males and females were separated and kept at 27°C, 70% relative humidity and a day/night (14:10 h) photoperiod cycle. Subsequently, females were paired with males in a courtship chamber (Meiselman et al., 2017). Following mating over 12 h, each female was isolated in a small round inverted cup (6 cm in diameter, 10 cm high) with inside a tube (tie 16 holes) filled with 4 ml of orange juice. The numbers of eggs laid by successfully mated females were recorded for three consecutive days.

### Rescue of RNAi Effect by Injection of 20E or Methoprene

To rescue the RNAi effect by ds*ETH* and ds*ETHR*, we co-injected 20E (stock 5 μg/μl) and dsRNA (20E: 0.5 μg/fly; dsRNA: 1.5 μg/fly) or methoprene (stock 5 μg/μl, Sigma-Aldrich), and dsRNA (methoprene: 0.5 μg/fly; dsRNA: 1.5 μg/fly) into 12-day-old virgin females following mating with normal virgin males. The RT-qPCR data and egg production were analyzed as described above.

### Statistical Analysis

All statistical analyses were performed in SPSS 20.0. A Student’s *t*-test was used to determine the significance of differences between the treatment and control in the dsRNA injection and rescue assay. Means ± SE (standard error) were determined based on four biological replications.

## Results

### Localization of ETH in Inka Cells at the Female Tracheae

**FIGURE 1:**
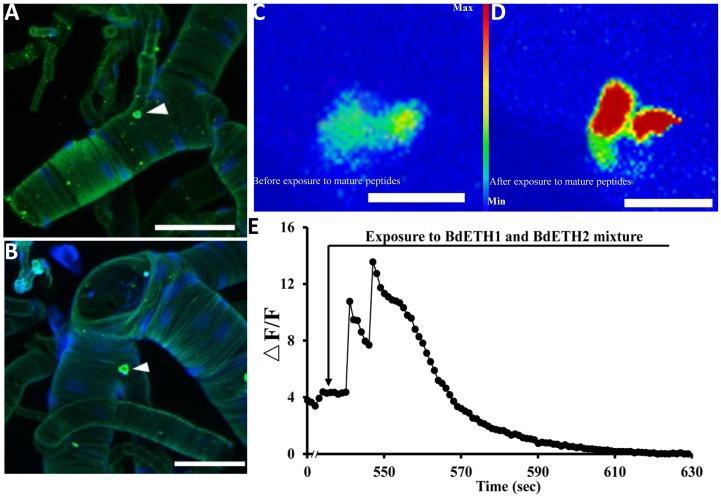
Localization of Inka cells in trachea of the female adult. White triangles indicate Inka cells stained using immunohistochemistry and *in situ* hybridization. **(A)** Trachea was dissected from a 5-day-old female adult and stained with an antiserum to DmETH1. **(B)** Trachea of a 15-day-old female adult stained with *in situ* DNA probe against *BdETH* mRNA. **(C,D)** BdETH mature peptides mobilizes calcium in the CA of female adult. CA from a female before **(C)** and after **(D)** exposure to 600 nM BdETH mixture peptides. **(E)** Time course of calcium mobilization in the CA in the response to 600 nM BdETH mixture peptides. Scale bar, 100 μm.

As shown in Figure 1A, we localized the Inka cells that produce/contain ETH peptide at the tracheae of the female adult of *B. dorsalis* by immunohistochemistry. Also fluorescent *in situ* hybridization by digoxin-labeled DNA probes with dissected tracheae of the 15-day-old females confirmed mRNA expression of *ETH* (Figure 1B). The adult Inka cells were localized at each branch point of the transverse connectives position of the female tracheae.

### Activation of the CA by Exposure to ETH *in vitro*

We dissected adult CA from 15-day-old female (Supplementary Figure S1), and immediately upon isolation, the calcium imaging was captured. The exposure to ETH (600 nM) produced a robust calcium mobilization, confirming activation of the CA by ETH (Figure 1C–E).

### cDNA Library and Expression Patterns of ETH Signaling in Female Adult Flies

**FIGURE 2:**
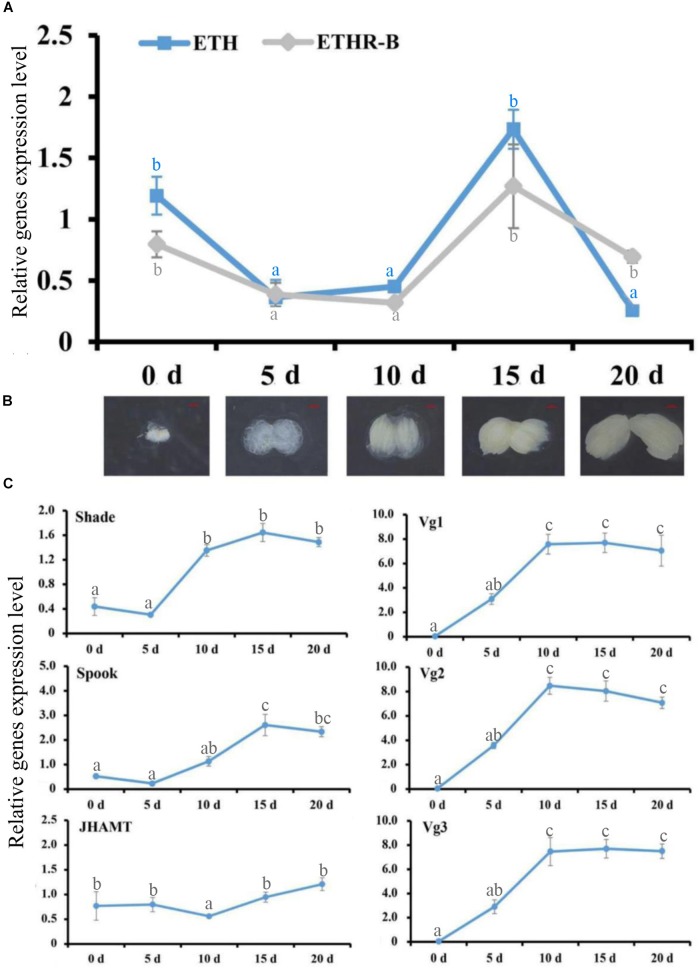
Ecdysis triggering hormone (ETH) signaling persists into the adult stage, evidenced by RT-qPCR. **(A)** Expression patterns of *BdETH* and *BdETHR-B* of female on days 0, 5, 10, 15, and 20 after eclosion detected by RT-qPCR. **(B)** The ovarian development including the day 0, 5, 10, 15, and 20 in the female adult of *B. dorsalis*. **(C)** Expression patterns of *Shade*, *Spook*, *JHAMT*, *Vg1*, *Vg2*, and *Vg3* detected by RT-qPCR in the female adult on days 0, 5, 10, 15, and 20 after eclosion. Different letters on the bars of the histogram indicate statistical differences based on the one-way ANOVA followed by Tukey’s honest significant difference (HSD) multiple comparison test (*P* < 0.05).

With the cDNA library of female adults at 0, 5, 10, 15, and 20 days after eclosion, the RT-qPCR profiles of ETH and ETHR-B showed the similar pattern (Figure 2). The expression of both genes started from a peak at the day of adult eclosion (day 0), then dropped to a basal level, and further increased again between day 10 and 15, whereafter it dropped to a basal level at day 20. In contrast, *ETHR-A* was absent over the whole period because the Ct values were ≥33; therefore it is not shown in Figure 2. In addition, the expression profiles of the two Halloween genes *Spook* and *Shade*, and the three vitellogenins Vg1, Vg2, and Vg3 showed the increased levels at days 10 and 15 (Figure 2C). On *JHAMT*, this was cloned and the phylogenetic analysis confirmed that *BdJHAMT* is closely related to *JHAMT* from other Diptera, such as *D. melanogaster*, *Glossina morsitans*, *Lucilia cuprina*, and *Aedes aegypti* (Supplementary Figures S2A,B). The expression of *JHAMT* showed a rise at day 15 which concurs with the increased levels of ETH and ETHR-B at that time-point (Figure 2C).

### 20E Regulates ETH Signaling in Female Adult

**FIGURE 3:**
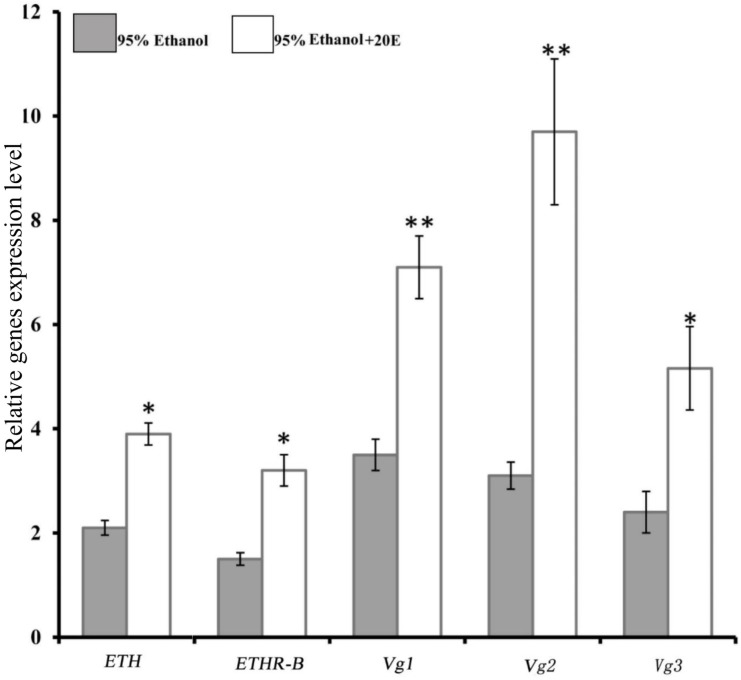
Induce of 20E signaling affect the expression of *BdETH*, *BdETHR-B*, *Vg1*, *Vg2*, and *Vg3* genes. Fold change in expression at 8 h after 20E injection measured by RT-qPCR in female adult of *B. dorsalis*. Data are means ± SD. Asterisks indicate significant differences with relative expression (^∗^*P* < 0.05; ^∗∗^*P* < 0.01). *BdETHR-A* was absent as the Ct values were >33.

We examined whether the expressions of *ETH* and *ETHR* are induced by 20E in the female adult of *B. dorsalis*. Indeed, with the injection of 0.5 μg of 20E into a 5-day-old female adult, which is a time moment of low basal levels of *ETH* and *ETHR-B* (Figure 2A), significant increases of the expression of *ETH* and *ETHR-B* were observed (Figure 3). Whereas *ETHR-A* stayed absent as Ct values were ≥33. 20E also plays a major role in significant promoting the *Vg1*, *Vg2*, and *Vg3* genes expression (Figure 3).

### RNAi of ETH Signaling Reduced Egg Production

**FIGURE 4:**
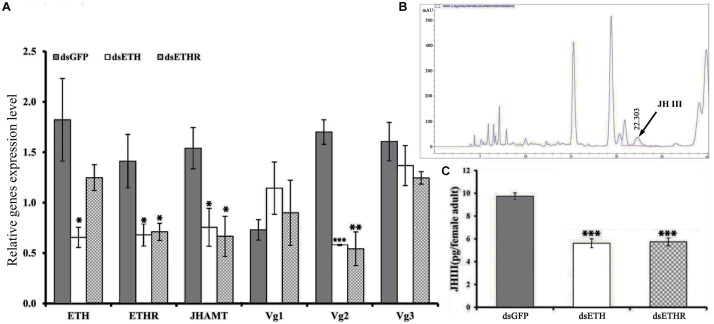
Silencing efficiency of genes of the ETH signaling (*BdETH/BdETHR*) in the female adult. **(A)** *BdETH*, *BdETHR*, *BdJHAMT*, *BdVg1*, *BdVg2*, and *BdVg3* transcript levels after silencing with RNAi compared with control. **(B)** HPLC spectrum of crude extract and JH III peak is detected in 22.303 min. **(C)** The column chart represents the JH III titer per female adult. Data are means ± SD. Asterisks indicate significant differences with relative expression (^∗^*P* < 0.05; ^∗∗^*P* < 0.01, ^∗∗∗^*P* < 0.001).

At 24 h after injection of specific dsRNA, the knockdown efficiencies for *ETH* and *ETHR-B* were 62 and 56%, respectively (Figure 4 and Supplementary Figure S3). With dsETHR injection, *ETHR-A* stayed absent as Ct values were ≥33 (Supplementary Figure S3). In these RNAi-ETH females, we observed a significantly decreased expression of *ETHR*, *JHAMT*, and *Vg2* (*P* < 0.05), whereas the expressions of *Vg1* and *Vg3* were unaffected (Figure 4A). However, in RNAi-ETHR females, we observed a decreased expression of *JHAMT* and *Vg2*, but not of ETH, *Vg1* and *Vg3* (Figure 4A). As *JHAMT* was decreased, we measured the JH III titers of female adult by HPLC. With an optimized HPLC protocol using, a gradient elution procedure with a total run time of 30 min to quantify JH III (22.303 min), we confirmed a significant decrease of the JH III titer by 50% and this was the case for ds*ETH* and ds*ETHR* (Figure 4B,C).

### Rescue of RNAi Effects by Injection of 20E and JH Mimetic (Methoprene)

**FIGURE 5:**
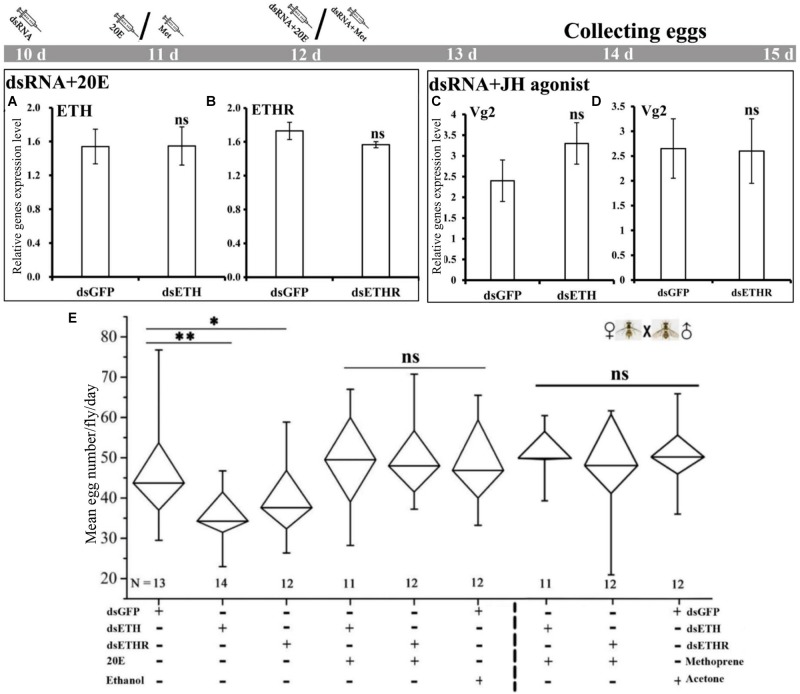
Impairment of ETH signaling resulted in reduced reproduction and 20E or methoprene injection rescued the egg production in female adults of *B. dorsalis*. **(A–D)** represent *BdETH*, *BdETHR*, and *Vg2* expression levels. **(E)** Represents the mean egg numbers per fly per day for the mated and RNAi female adults. The Whisker plot shows the maximum, minimum, and median values. dsGFP, double stranded green fluorescent protein; dsETH, double stranded ecdysis triggering hormone; dsETHR, double stranded ecdysis triggering hormone receptor. NS represents no significant difference; ^∗^*P* < 0.05; ^∗∗^*P* < 0.01, *t*-test.

The co-injection of 20E into the RNAi-females with a knockdown of *ETH* or *ETHR*, could rescue the expression of *ETH* and *ETHR-B*, and also the egg production was increased to normal levels as in the control groups (Figure 5A,B,E). Also the injection of the JH mimetic methoprene into these RNAi-females could rescue the expression of *Vg2* and egg production (Figure 5C–E). With dsETH injection females, we observed a significant decrease in egg production (*P* < 0.01). In these RNAi-ETHR females, the results also showed a significant dropping in egg production (*P* < 0.05) (Figure 5E).

## Discussion

In this study, we confirmed the presence of Inka cells and ETH in the female adult of *B. dorsalis* by immunohistochemistry and *in situ* hybridization. In the larval stages of *B. dorsalis*, the Inka cells are positioned just at each branch point of the transverse connectives of the tracheae (Shi et al., 2017). This result is in agreement with the observations in *Drosophila* (Meiselman et al., 2017; Lee et al., 2017). As seen also in *Drosophila* (Meiselman et al., 2017), the expression of ETH and its receptor are in phase with growth of the ovary. The correlation of ETH with ovary growth will be discussed later (Figure 2B). Interesting in our work is the *ETHR-B* receptor that is present in the female adult stage, while *ETHR-A* is absent with Ct values of ≥33. This is in great contrast to the presence of ETH signaling during the larval stages of *B. dorsalis* that is involved in successful shedding of the cuticle for molting where the *ETHR-A* was present (Shi et al., 2017). At present we have no deeper insights in the reasons of this unique difference for the two ETH receptors, where ETHR-B seems to have a role in the female adult in reproduction regulation versus *ETHR-A* in the larval molting process. Future research can focus on factors as tissue specificity, functional role in larval versus adult stages, ligand binding sites specificities. However, differences between fruit flies with *D. melanogaster* that is the super model for scientific research, and the less studied *B. dorsalis* that is of large agriculture importance, may occur as was also reported before for other peptide hormones (Gui et al., 2017).

On the role of ETH in the female adult of *B. dorsalis*, our data agree with previous reports that ETH signaling plays a vital functional role as an allatotropin for maintenance *JHAMT* expression required for normal JH, vitellogenesis and reproduction in females (Meiselman et al., 2017). Indeed, when we incubated the CA tissues from females with ETH peptide, CA was activated. Also in the RNAi assays with dsETH-ETHR injection, the *JHAMT* was downregulated, and the JH titers and the egg productions were all reduced. These data suggest that receptors for ETH peptide are present in the CA of *B. dorsalis* adults, implying that this endocrine gland is the target of ETH. In previous studies, ETHR transcripts were detected in the CA of 4th and 5th instar larvae of *Bombyx mori* (Yamanaka et al., 2008). Also intriguing here is that the knockdown of *ETH* and its receptor via RNAi led to a reduction of *Vg2* only, but not for *Vg1* and *Vg3*. The reason for this we believe is that *Vg2* had a great response to JH, whereas *Vg1* and *Vg3* are more responsive to variation in ecdysone signaling (Chen et al., 2012; Tarone et al., 2012). Taken together, our data confirmed that loss of the ETH signaling impaired the JH levels and vitellogenin synthesis, and then restrained the egg production in the female flies.

On JH, it is well known that this sesquiterpenoid hormone regulates the female fertility by controlling the biosynthesis of vitellogenin and its uptake by the growing oocyte (Jindra et al., 2013). This was reported in ants (Brent et al., 2006), wasps (Dong et al., 2009), bees (Pinto et al., 2000), beetle (Parthasarathy et al., 2010b), and *Drosophila* (Marchal et al., 2011). JH III is found in all insects, except for the Hemipteran (Kotaki et al., 2009; Bendena et al., 2011; Meiselman et al., 2017). Therefore, we believe that also in *B. dorsalis* it is JH III that regulates the reproductive maturation in the female adult. Our RNAi experiments resulted in a reduction of *JHAMT* and the JH titer (Figure 4B,C), and this led to a loss of egg production.

We confirmed with this work in *B. dorsalis* that the steroid hormone 20E is an upstream regulator for ETH expression and its receptor. At the larval stage of *Drosophila*, rising of the ecdysteroid levels resulted in an increase of ETH levels and size of the Inka cells (Kingan et al., 1998). This agrees with our observations that the expression of both *ETH* and *ETHR-B* showed an increase between day 10 and 15, and this co-occurred with the peak levels of the two Halloween genes *Spook* and *Shade*, which were involved in 20E production at days 10–15 after adult eclosion (Figure 2C). Besides, injection of 20E in 5-day-old females increased the expression of *ETH* and *ETHR-B*, and moreover the injection of 20E in RNAi-females could rescue the expression of ETH, its receptor and reproduction (Figure 5).

## Conclusion

In conclusion, we investigated the role of the ETH/ETHR pathway in the reproduction of *B. dorsalis*. It was of interest that it is the ETHR-B isoform that is playing a role in the female adult via JH in ovary growth regulation. This is in great contrast to the larval immature stages of *B. dorsalis* where it is the ETHR-A isoform that is regulating for successful shedding of the cuticle for molting (Shi et al., 2017). Hence, with RNAi and rescue experiments, we showed here that ETH signaling is under control of a pulse of 20E. With this information, we also shed light on potential novel insecticidal targets for instance with use of hormone antagonists to control important pest insects.

## Ethics Statement

The research project was conducted on invertebrate species that are not subjected to any specific ethical issue and legislation.

## Author Contributions

YS, H-BJ, YP, WD, YP, and GS designed the research. YS, T-YL, and X-QL performed all of the experiments with the help of H-BJ. YP, GS, and J-JW provided the materials. YS, H-BJ, YP, GS, and J-JW analyzed the data. YS, H-BJ, YP, GS, and J-JW wrote the manuscript.

## Conflict of Interest Statement

The authors declare that the research was conducted in the absence of any commercial or financial relationships that could be construed as a potential conflict of interest.

## Footnotes

1 https://i5k.nal.usda.gov/Bactrocera_dorsalis
