# Supplementary material for: The Ecdysis Triggering Hormone System, via ETH/ETHR-B, Is Essential for Successful Reproduction of a Major Pest Insect, Bactrocera dorsalis (Hendel)
Source: Front Physiol. 2019 Mar 18;10:151. doi: 10.3389/fphys.2019.00151 (PMC6431669; doi:10.3389/fphys.2019.00151)
Supplement: Supplementary file 1 [file Data_Sheet_1.PDF]

The ecdysis triggering hormone system, via ETH/ETHR-B, is essential for successful reproduction of a major pest insect, *Bactrocera dorsalis* (Hendel)

Yan Shi[1,2], Tian-Yuan Liu[1,2], Hong-Bo Jiang[1,2], Xiao-Qiang Liu[1,2], Wei Dou[1,2], Yoonseong Park[4], Guy Smagghe[1,2,3*] and Jin-Jun Wang[1,2*]

[1]Key Laboratory of Entomology and Pest Control Engineering, College of Plant Protection, Southwest University, Chongqing 400716, China.

[2]Academy of Agricultural Sciences, Southwest University, Chongqing 400716, China.

[3]Department of Plants and Crops, Faculty of Bioscience Engineering, Ghent University, Ghent, Belgium.

[4] Department of Entomology, Kansas State University, Manhattan, KS 66506.

*** Correspondence to: Dr.** Guy Smagghe (guy.smagghe@ugent.be) and **Dr.** Jin-Jun Wang (wangjinjun@swu.edu.cn or jjwang7008@yahoo.com), College of Plant Protection, Southwest University, Chongqing 400716, P. R. China.

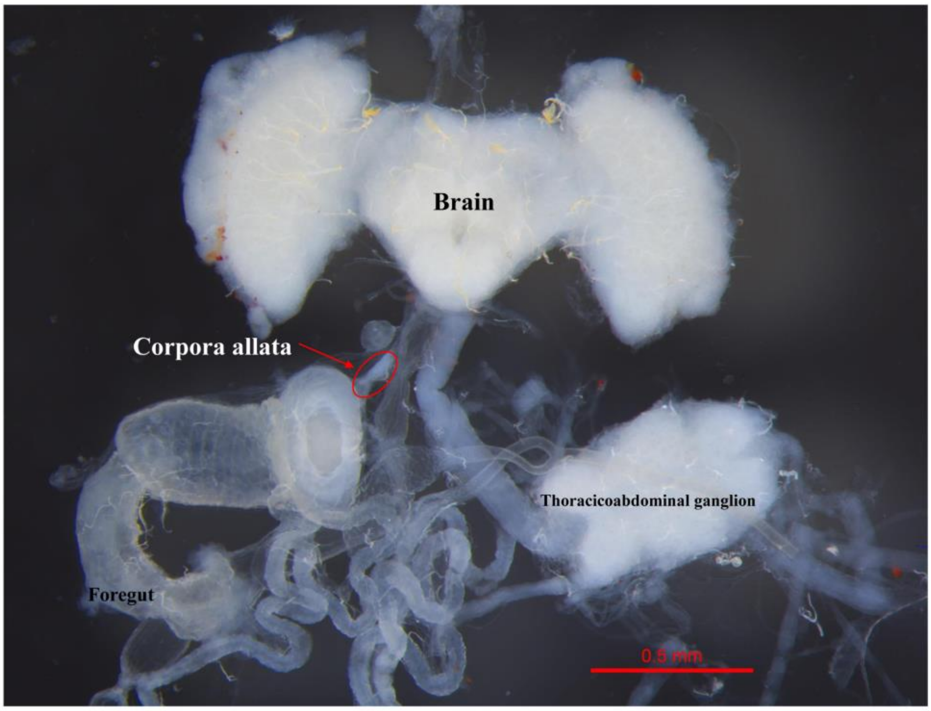

Figure S1. Dissected tissues including brain, CA, esophagus, gut and thorax ganglion on the 15th day post-eclosion of female adult of *B. dorsali*s. Scale bar, 0.5 mm.

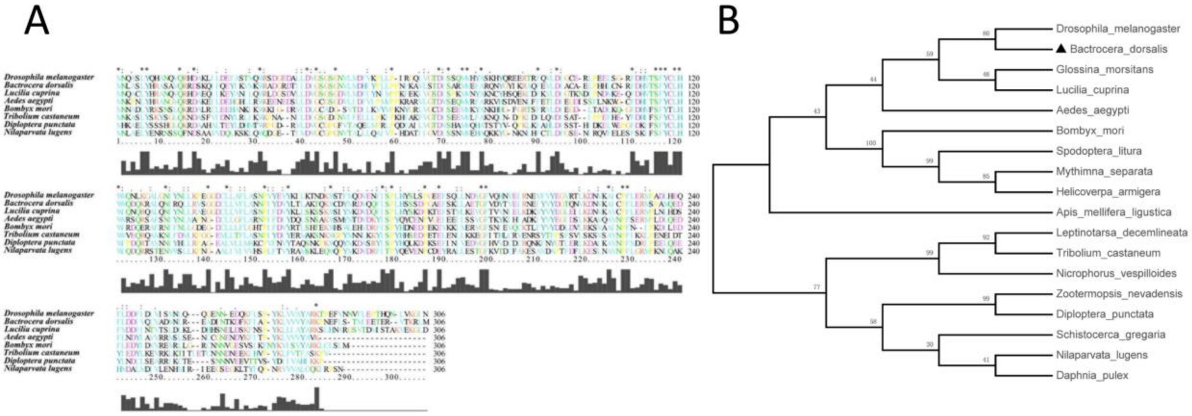

Figure S2. Multiple alignment of eight insect JHAMT and phylogenetic analysis of JHAMT homologs from different insect species based on amino acid sequences.

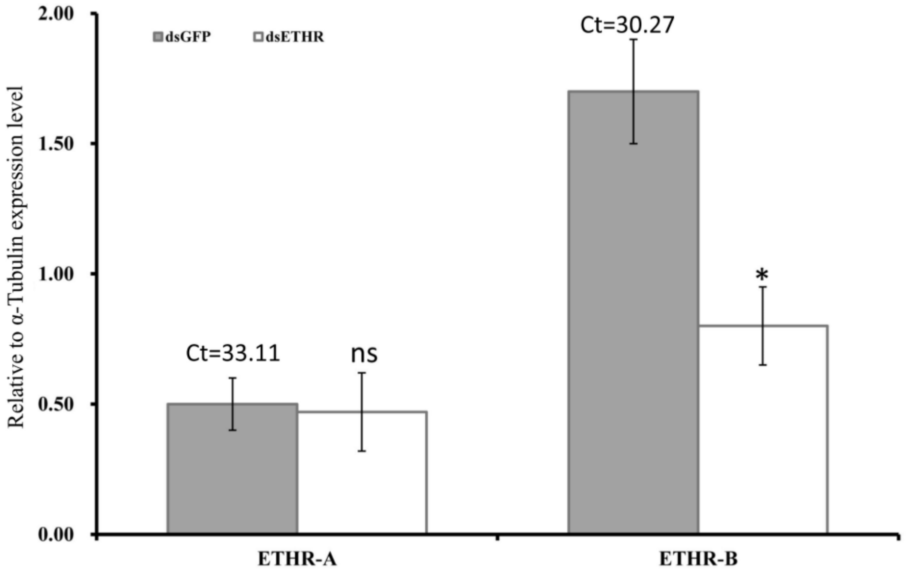

Figure S3. Silencing efficiency of *BdETHR* on the expression of its receptors in female of *B. dorsalis*. *BdETHR-A* and *BdETHR-B* transcript levels in female after RNAi compared with control. NS represents no significant difference; *, $P < 0.05$, t-test.

Table S1. Primer sequences used in this study

Target	Direction	Sequence 5' to 3'
BdJHAMT (Cloning)	Forward	ATGAATCTCGCATCGCTTTATCAC
	Reverse	TCACATCAAACGCTTTGTCACACG
BdETH (qRT-PCR)	Forward	CGTAAACCGGATCAACGACT
	Reverse	GCGCATAAAGTCTCCACCAT
BdETH-R-A (qRT-PCR)	Forward	GATTGGCGTCGAACGTTACT
	Reverse	ATAGCGTCCGCAGGTTATTG
BdETH-R-B (qRT-PCR)	Forward	CATTCTGGTCGGCATTCTTT
	Reverse	ATTGTTGGCGCAAGATAACC
BdVg1 (qRT-PCR)	Forward	CAACCAATCAGCAATAACCAGGAC
	Reverse	GCATCACCACGAGCCAAACC
BdVg2 (qRT-PCR)	Forward	CAGGAAGGAGAGCGTTTGATTGG
	Reverse	CTGTTGTCCGTAGTAGCGTTGC
BdVg3 (qRT-PCR)	Forward	CCCAGTCCCAGCGATATTCC
	Reverse	ACTTGTCCGTTGTAGGCCTG
BdETH (dsRNA)	Forward	taatacgactcactatagggCCATTGTCAGGGCAATGAA
	Reverse	taatacgactcactatagggGTTGTAATATATATAATCG
BdETHR (dsRNA)	Forward	taatacgactcactatagggTCTTATGTACGCAACACAGC

	Reverse	taatacgactcactataggg GGTGGCGTATTTACCTCGAC
GFP (dsRNA)	Forward	taatacgactcactatagggCAGTTCTTGTTGAATTAGATG
	Reverse	taatacgactcactatagggTTTGGTTTGTCTCCCATGATG
Bd-specific probe *ETH*	Forward	ACATTTGGCGTTTTGCTCTT
(*situ* hybridization)	Reverse	CTTCGGCTGGATTCATAAGC
α-Tubulin (internal reference)	Forward	CGCATTCATGGTTGATAACG
	Reverse	GGGCACCAAGTTAGTCTGGA

Table S2. Sequences and relevant information used for phylogenetic analysis of the insect JHAMT

Species	GenBank No.
Leptinotarsa decemlineata	KP274881
Bombyx mori	JN851817
Drosophila melanogaster	NM_001299051
Daphnia pulex	AB222845
Zootermopsis nevadensis	LC049616
Mythimna separata	KM926339
Apis mellifera ligustica	KC335148
Glossina morsitans	JX291120
Schistocerca gregaria	HQ634702
Helicoverpa armigera	AB127945
Spodoptera litura	AB127944
Tribolium castaneum	NM_001127311
Lucilia cuprina	KNC28768
Aedes aegypti	XP_001651876
Nicrophorus vespilloides	XP_017772157
Diploptera punctata	AHZ20738
Nilaparvata lugens	AKU04654
Bactrocera dorsalis	MG269999
